# On a New and Successful Mode of Treating Extensive Abscesses

**Published:** 1818-04

**Authors:** John Cunningham

**Affiliations:** Surgeon of His Majesty's Ship Rochefort


### *On a new and successful Mode of treating* EXTENSIVE ABSCESSES. By JOHN CUNNINGHAM, Surgeon of His Majesty's Ship Rochefort.

AS the last eighteen years of my service, in his Majesty's navy, have been in ships of the line, it will scarcely be doubted that the disease in question must have frequently been the subject of observation and treatment; especially when we consider the liability of sea-faring men to wounds, bruises, and abrasions, on the extremities; the slighter cases of which are too frequently neglected by the patients themselves, until the parts begin to inflame and fester, and till the absorption of vitiated matter occasions, very often, inflammation and tumefaction of the contiguous lymphatics and glands, which commonly form the site of large and troublesome abscesses.

When the remedies generally employed for the dispersion of inflamed tumours have proved ineffectual, and they have passed into the suppurative stage, and become matured abscesses, surgeons have established three modes of treatment; viz. to give vent to the matter by the application of caustic; by incision with the knife; or the introduction of a seton. I shall not presume to animadvert upon the comparative advantages or disadvantages of these modes of treatment; they are self-evident to every practitioner. Two circumstances of importance, in either way, have been justly regarded, and their pernicious consequences detailed; namely, the admission of the atmospheric air into the cavity of the abscess, occasioning gangrene, &c.; and the danger to which the constitution of the patient is exposed, from long continued discharge, or absorption, of matter from the diseased surface.

The mode of treatment which I beg leave to suggest, and which I have practised with invariable success for several years, obviates these evils entirely, and will be found a speedier and safer way of obtaining a cure, than by any other practice to my knowledge. The tedious cures which I often observed my patients subjected to under the usual manner of treatment, in spite of all the care and attention I could pay, grieved me, and occasioned a series of reflections on the subject. The result was, a determination to try whether adhesion, by the first intention, after fully evacuating the contents of the abscess, and afterwards replacing the integuments *in situ*, might not, even with a diseased surface, be obtained.

A case soon afterwards occurred, every way suitable to my views. It was a large abscess on the knee, containing full half a pint of thinnish, streaky matter. With a scalpel, I laid it open at once, by a longitudinal incision over the centre of the patella, beginning and ending on the sound skin. I squeezed all the matter as clearly out from every part of the cavity, which descended very low on each side of the joint, as I possibly could (not regarding the access of air into it); I then brought the lips of the incision into contact, as in a common incised wound, and secured them in that situation by adhesive straps, closely applied, in the usual manner. I next placed compresses on each side of the knee, so as to afford equal pressure on every point, from the most inferior parts of the sac towards the incision; these were secured by an eighteen-tailed bandage, properly adapted, and the whole dressings were wetted by a cold lotion. The limb was placed extended in bed; and to prevent the possibility of flexion, a long splint was placed under the ham, and secured in that situation. An opiate was administered.

On the third day afterwards, the dressings were removed, when, to my satisfaction, I found that perfect adhesion of the integuments to the parts beneath was obtained throughout; the lips of the wound were in close contact and united; not a vestige of discharge was to be seen; he had enjoyed perfect exemption from pain, except a trifling uneasiness from the extended state of the limb, and otherwise he felt comfortable. Dressings were re-applied as before, and the splint and lotions continued for a couple of days longer. The cure went on without interruption, and on the 18th day from the operation he returned to his duty; his knee being perfectly sound, exhibiting only a cicatrix like a line over the patella; and from his insertion on the sick-list, until his discharge, he was only twenty-seven days from duty, being entered on the 12th of June, had the abscess opened on the 21st, and he returned to his duty on the 9th of July following. In many instances since, I have obtained the same salutary result invariably. The three last cases, as the subjects of them still belong to the Rochefort, I shall briefly detail as they stand on my note book.

Portsmouth Harbour, November 10th, 1815. William Reilley, ætat. 29, had a few days previously joined the ship, and complained of an abscess between the first and middle fingers of his left hand. The superficial lymphatics of the fore-arm were already in a state of inflammation and enlargement, and a painful tumour, of considerable size, was forming above the bend of the arm, extending upwards over the brachial artery about four inches. A repellent lotion was employed to the tumour, and an emollient poultice to the hand, which soon ripened the abscess between the fingers, which was opened, and by the 20th it had become a clean granulating sore. The tumour on the arm, in the mean time, had arrived at considerable magnitude, and was evidently disposed to suppurate; the cold lotions were therefore discontinued, and poultices, frequently renewed, applied instead.—Dec. 1st. Fluctuation evident throughout the sac. A longitudinal cut, about five inches in length, rather in an oblique direction, to favour the entire escape of the matter, was made through the centre of the tumour, beginning and ending on the sound substance. A considerable quantity of matter was discharged, leaving a very hollow cavity, and the remainder was carefully squeezed out. The lips of the incision were brought closely together, and secured by adhesive straps round the arm; a compress was placed over the site of the abscess, and secured by a roller, affording uniform support both ways; and the dressings kept constantly moistened with the cold saturnine lotion, opiates, &c. as circumstances required.—3d. No pain worth noticing since the opening of the tumour; dressings removed: a slight discharge from the lips of the wound, which have not united; but the integuments have, throughout, adhered firmly to the cavity of the abscess. Dressed as before.—6th. Discharge much diminished. Edges of the wound have approximated, and appear as a mere line along the integuments.—14th. Lips of the wound a little callous; apply argent. nitr. and dressings as before.—20th. Wound completely healed; sore between the fingers nearly so.—26th. The latter also healed, and he has returned to his duty.

John Phillips, aged about 37, a native of France, complained, on the 11th of March, 1816, of a large colourless swelling on his left elbow-joint, which had been some time in arriving at the state in which I saw it, the cause of which he could not account for; nor was there any sore, or appearance of injury on the hand, which might likely occasion such a complaint. It had become very painful; and, besides the tumefaction over the olecranon, the swelling extended about three inches lower down the arm, in the direction of the ulna. Fluctuation of matter was evident: an emollient poultice was therefore applied to the tumour at once, and frequently renewed; and a purge was a dministered. Next day, the abscess appearing fully ripe for opening, an incision was made with a scalpel through the integuments its whole extent. The matter, which was thin and streaky, and in considerable quantity, was well cleared out from the sac. The lips of the incision were closely approximated, and secured by adhesive plasters and otherwise treated, as in the preceding case.

14th. Dressings removed. Cavity of the abscess completely obliterated; incised parts only not closed. Dressed and treated as at first.

23d. Wound almost closed, except at the inferior extremity, where the lips are callous; and it is necessary to remark here, the absolute necessity of commencing and terminating the incision on sound substance: likewise, of making the compression on every part of the cavity from its most distant extremity, in every direction, towards the centre, uniform and steady; otherwise, matter may collect behind, and totally defeat our purpose. It was the case in this instance. The termination of the incision was not carried low enough by some lines; increased action commenced at the extremity, where the diseased substance was not fully divided; the parts below, for a considerable distance, became inflamed, which, in spite of repellent remedies assiduously applied, terminated in another abscess of considerable size. It was ripened by poultices, and fit for opening on the 30th. As the upper boundary of the sac communicated with the lower end of the incision, a director was easily introduced from thence into it, and pushed to its most depending part, and the integuments divided to the bottom with the sharp pointed bistoury; so that, in this case, by the second opening, they were divided from a little above the olecranon ulnæ, to within two inches of its carpal extremity, in the direction of the bone. Treatment as formerly; and on the 14th of next month he was discharged to his duty, his arm being perfectly sound, and has continued so since.

The third case affords still more satisfactory evidence of the advantage of the practice.—James Reilley, ætat. 22, being employed in the Camelion, one of the ship's tenders, and carrying a jar of beer down a ladder, fell, and broke it. A fragment cut his arm transversely, in two places, a little below the olecranon, pretty deeply, which occasioned some surrounding inflammation. Before the wounds were quite healed, while assisting in clearing the vessel's bottom, on

Gosport beach, one of her legs gave way, and she fell over; by which accident he received a violent contusion, a little anterior to the former injury. Very high inflammation and tumefaction supervened along the whole forearm, which, in spite of the most diligent application of cold repellent lotions, terminated in the formation of a large abscess. Warm cataplasms were now employed to hasten it to maturity; and on the 3d instant, it was opened in the following manner:—The point of a bleeding lancet was introduced obliquely from the sound skin into the upper edge of the sac, which was nearly over the head of the radius. A little matter escaped from the puncture. A director was introduced at the opening, and conveyed to the most depending part of the tumour, a distance of fully five inches, in the direction of the extensor digitorum communis (the transverse diameter was fully four inches). With the sharp-pointed bistoury, the integuments were divided to the bottom, until the point emerged from the sound surface, whereby the sac was nearly divided into two equal parts. Upwards of six ounces of thick matter were discharged, leaving a large cavity with deep indentations among the muscular substance; and the numerous bridles of cellular membrane connected with the integuments, and between the muscles themselves, which intersected the cavity in every direction, exhibited a very curious appearance. The matter being cleared out, as completely as possible, from every part of the sac, the lips of the wound were approximated; pads of lint and linen, corresponding to the depth and extent of the cavity, were placed on each side of the incision, and secured by adhesive straps; and as the lower part of the arm and hand were much swollen, and very œdematous, a roller was applied from the fingers to the middle of the humerus, and again to the wrist, in opposite directions, affording regular support to every point, and the whole wetted with cold water, frequently renewed. Had an opiate, and was allowed to rest as he inclined through the day.

4th. Slept all last night soundly, having been free from pain, besides having scarcely shut his eyes for a week before, by reason of the excessive pain he endured. Is perfectly easy; no appearance of discharge. Lotion continued; dressing removed. Cavity completely obliterated; the wound has retracted a little in the centre, but is filled with healthy granulations; a very trifling discharge. Incised parts joined by next application of the straps; bandage and lotion, as before. The œdema of the metacarpus disappeared within the first twenty-four hours. The interposing granulations being destroyed by the application of caustic, the edges of the wound daily approximated; and on the 22d, the skin was closely united, exhibiting a cicatrix like a mere line.

On the 24th, he returned to his duty, feeling no inconvenience from the injury.

From the facts above stated, a train of practical reflections arise, not only in regard to the management of abscesses, but with respect to the treatment of sinuous ulceration, which the limits of a paper do not permit me to indulge in. So far, I beg permission merely to glance, as briefly as possible, at a few of the most obvious and important of the advantages of the mode of treatment inculcated. By the above method, all the ill consequences described by authors are obviated; for by approximating the lips of the wound immediately after the contents of the sac are discharged, and applying the dressings as I have described, the external air (if it really be productive of the evils supposed) is effectually excluded; adhesion and obliteration of the sac will certainly be obtained; the health of the patient will be nowise injured, either by the quantity or quality of the subsequent discharge; he has next to entire exemption from pain during the cure; as the cutis vera approximates closely, when the cure is effected, there is no waste of cutaneous substance, which frequently renders the parts weaker afterwards than they were before; there will be no unseemly or puckered cicatrices, so often observed on the site of large abscesses: and, finally, of not the least importance, instead of the curative process occupying weeks, nay, months sometimes, by the plan I have adopted, the cure is almost effected in a few days; for when the cavity of the abscess is once obliterated, by the adhesion of the investing integuments to the surface below, I consider the cure as next to completed, as the lesion then constitutes merely a simple wound.*

I am, &c.

JOHN CUNNINGHAM, Surgeon.

H.M.S. Rochefort.

*Portsmouth Harbour,*

*Nov.* 29, 1817.

P. S. Since the above statement was drawn up, three

* Dr. Johnson, one of the Editors, can bear witness to the last case detailed.

additional cases have occurred, terminating, by the same treatment, in the same salutary manner. One was a pretty large abscess in the axilla, evidently produced by absorption from two bruised sores on the fingers, already perfectly healed. Another, rather a large tumour, on the shin of a sickly lad, occasioned by no obvious cause. When it was opened, two inches of the entire front of the tibia were exposed, perfectly denuded, except by the periosteum, which appeared as white as flax. The third, an abscess occupying the whole front of the knee of a woman, over the ligament of the patella, occasioned by a violent contusion on the part.

